# Pregnant People-Infant Linkage for Surveillance

**DOI:** 10.23889/ijpds.v9i5.2635

**Published:** 2024-09-18

**Authors:** Amro Hassan, Andrew Hamilton, Shelly Sital

**Affiliations:** 1 AllianceChicago

## Objectives and Approach

As maternal mortality and morbidity continue to progress, better data is needed to comprehensively understand the problem, especially in underserved populations who are differentially impacted. Surveillance in safety-net populations is complicated by care fragmentation and interoperability challenges between primary care and hospital settings. . AllianceChicago (AC), a health center-controlled network, supports a common data infrastructure across a set of Federally Qualified Health Centers (FQHCs) in Chicago that is further linked to a distributed query infrastructure connecting Chicago’s major health systems known as CAPriCORN.

Leveraging electronic health record (EHR) data collected by the AC and CAPriCORN consortium, the objective of this surveillance effort was to connect infant data with maternal data while protecting privacy. We developed a deterministic matching algorithm that utilized multiple approaches and technologies for Pregnant People-Infant (PPI) pair identification. The linkage process involved a five-step method in which each step is recorded within the linkage database and the confidence interval is calculated.

## Methods

We analyzed three chart review cohorts (4,008 patients in 2003, 3,045 in 2015, and 9,024 in 2022) in Calgary, Canada. Nurse reviewers determined the presence or absence of 17 clinical conditions employing similar protocols. The reviews were linked with DAD using a unique lifetime identifier, chart number, and admission date. We evaluated the validity of DAD, coded in ICD-10-Canada version, in recording conditions by comparing against chart reviews. The C-statistics was calculated in predicting in-hospital mortality.

## Results

Preliminary analyses are ongoing. Through each step of the linkage process, the confidence interval increased with the increase of matching nodes, highlighting its accumulative accuracy. The result demonstrates the feasibility of linking maternal and baby healthcare characteristics using a range of clinical and demographic variables captured in pseudonymized hospital and ambulatory data.

## Conclusions and Implications

Our approach improves the capacity of EHR data for surveillance while protecting patient privacy and preserving data confidentiality. Triangulating outcomes recorded in different settings can help improve data quality for research and surveillance.

